# Design and psychometric evaluation of epilepsy-related apathy scale (E-RAS) in adults with epilepsy: a sequential exploratory mixed methods design

**DOI:** 10.1186/s12883-021-02139-2

**Published:** 2021-03-17

**Authors:** Abbas Shamsalinia, Mozhgan Moradi, Reza Ebrahimi Rad, Reza Ghadimi, Mansoureh Ashghali Farahani, Reza Masoudi, Leili Rabiei, Fatemeh Ghaffari

**Affiliations:** 1 Nursing Care Research Center, Health Research Institute, Babol University of Medical Sciences, Babol, Iran; 2 Iran University of Medical Sciences, Tehran, Iran; 3 Department of Medicine, Islamic Azad University, Tonekabon Branch, Tonekabon, Mazandaran Iran; 4 Social Determinants of Health Research Center, Health Research Institute, Babol University of Medical Sciences, Babol, Iran; 5 Nursing Care Research Center and School of Nursing and Midwifery, Iran University of Medical Sciences, Tehran, Iran; 6 Community-Oriented Nursing Midwifery Research Center, Department of Adult and Geriatric Nursing, Nursing and Midwifery School, Shahr-e-Kord University of Medical Sciences, Shahr-e-Kord, Iran; 7 Social Determinants of Health Research Center, Shahrekord University of Medical Sciences, Shahrekord, Iran

**Keywords:** Factor analysis, Epilepsy-related apathy, Psychometric, Reliability, Validity

## Abstract

**Background:**

Apathy in patients with epilepsy is associated with a wide range of consequences that reduce the patient’s ability to perform social functions and participate in self-care and rehabilitation programs. Therefore, apathy is one of the important diagnoses of the healthcare team in the process of caring for epileptic patients and its dimensions need to be examined and recognized. Therefore, appropriate instruments with the sociocultural milieu of each community should be provided to health care providers. The aim of the present study was to design and measure epilepsy–related apathy scale (E-RAS) in adults with epilepsy.

**Methods:**

This study of sequential exploratory mixed methods design was conducted in Iran from April 2019 to December 2019. In the Item generation stage, two inductive (face-to-face and semi-structured interviews with 17 adult epileptic patients) and deductive (literature review) were used. In item reduction, integration of qualitative and literature reviews and scale evaluation were accomplished. For Scale Evaluation, face, content, construct [exploratory factor analysis (EFA) (*n* = 360) and confirmatory factor analysis (CFA) (*n* = 200)], convergent and divergent Validity and reliability (internal consistency and stability) were investigated.

**Results:**

The results of EFA showed that E-RAS has four factors, namely, motivation; self-regulatory; cognition and emotional-effective. These four latent factors accounted for a total of 48.351% of the total variance in the E-RAS construct. The results of CFA showed that the 4-factor model of E-RAS has the highest fit with the data. The results of convergent and divergent validity showed that the values of composite reliability (CR) and average variance extracted (AVE) for the four factors were greater than 0.7 and 0.5, respectively, and the value of AVE for each factor was greater than CR. The Cronbach’s alpha coefficient for the whole scale was obtained 0.815. The results of the test-retest showed that there was a significant agreement between the test and retest scores (*P* < 0.001).

**Conclusion:**

E-RAS is a multidimensional construct consisting of 24 items, and has acceptable validity and reliability for the study of epilepsy-related apathy in adult epileptic patients.

**Supplementary Information:**

The online version contains supplementary material available at 10.1186/s12883-021-02139-2.

## Highlights

- Epilepsy in adults is associated with several problems due to its chronic nature and symptoms such as seizures and social stigma.
- Disease-related problems may affect the patient’s understanding, feeling, and behaviour toward the disease and lead to behavioural symptoms in the patient.
- Apathy in patients with epilepsy can have a wide range of consequences and should be investigated and identified.
- To gain information on epilepsy-related apathy, appropriate instruments with the socio-cultural milieu of each community are needed.
- The E-RAS is a valid and reliable instrument for assessing the motivation; self-regulatory; cognition and emotional-effective dimensions of the apathy in adults with epilepsy.

## Background

Neurological disorders are one of the chronic diseases that are commonly associated with apathy. In the literature review, 20–80% of patients with Parkinson’s disease, progressive supranuclear palsy, stroke [1] and Alzheimer’s disease [2] were reported to have epilepsy. Apathy in patients with chronic diseases can reduce treatment response due to reduced adherence to the treatment protocol [3]. Therefore (For this reason), apathy due to cognitive and emotional problems associated with the disease in chronic disease patients (in patients with chronic diseases) has currently been emphasized [4].

Epilepsy is a chronic non-communicable brain disease that affects about 50 million people worldwide and therefore accounts for one of the leading neurological diseases across the world. The incidence rate of epilepsy is reported to be 61.4 per 100,000 population every year (95% CI; 50.7–74.4) [5]. The prevalence of epilepsy in Iran is reported to be 5% (95% CI: 2–8) [6]. Self-care behaviors are the basis for treating and controlling seizures that can be affected by apathy [7]. Although apathy can be a personality trait, suffering from a chronic illness may lead to the disease-related apathy due to long-term involvement with it and its treatment process. Apathy refers to a set of behavioural, emotional, and cognitive traits such as decreased interest in daily activities, lack of motivation to engage in creative activities, a tendency to withdraw early from activities, lack of interest, and diminished emotions [8] various definitions and characteristics have been provided for the concept of apathy. Some define indifference as a lack of motivation related to the patient’s level of performance in terms of age and culture, provided that lack of motivation in apathy is not due to decreased level of consciousness, cognitive impairment, emotional distress, or direct physiological effects of the use of substances such as drugs or medications [9].

Other researchers have referred to apathy as the failure to respond to stimuli in the form of inaction. Others consider apathy to be a disorder in the dimensions of executive cognition or will (i.e., a decrease in human power, potency, or ability to initiate action, or a low desire for goal formulation and voluntary behavior) [10, 11]. In clinical settings, apathy is diagnosed by decreased vitality, decreased self motivation and poor initiative [8], lack of interest in learning new things or new experiences, and decreased emotional response to effective changes in the course of treatment or failure to respond to positive or negative events [12]. According to Robert et al. (2018), four indicators of apathy in patients with brain disorders include 1- Reduced goal-directed behaviour compared to the patient’s previous level of performance, 2. Having two of the following three main symptoms: Behavioral/cognitive symptoms (decreased level of public activity, less persistence, decreased interest, personal wellbeing), Emotional symptoms (verbal or physical expressions, impact on others, emotional reactions to the environment, less spontaneous emotions, empathy) and Social symptoms (spontaneous social initiative, environmentally stimulated social interaction, relationship with family members, verbal interaction, homebound) for at least 4 weeks and continuously. 3- Clinically, the first and second indicators lead to disruption in personal, social, occupational, or other important areas of functioning and 4 - Lack of simultaneity of the first and second indicators with other clinical disorders such as physical disabilities, motor disabilities, substance use or environmental changes [13].

Apathy in patients with chronic disease is associated with a wide range of consequences; for example, it can cause the patient not to strive for daily activities and become dependent on others or it can reduce the patient’s quality of life [14]. Apathy increases the care burden of family caregivers [15] and reduces the patient’s ability to perform social functions and participate in self-care and rehabilitation programs [12]; the patient’s care-related needs including physical, mental and social care increase as well [2].

Because apathy is challenging to diagnose due to its similar characteristics to depression and requires its own diagnostic instruments [8], and apathy treatment is often complicated and difficult, and the available guidelines for therapists (clinical psychologists) are limited, it is necessary to take preventive measures to identify the factors effective on the treatment process and improve the outcomes of treatment. Apathy is one of the important diagnoses of the healthcare team in caring for epileptic patients, and therefore should be examined and characterized [12, 16, 17]. For this purpose, appropriate instruments with each community’s socio-cultural milieu should be provided to healthcare providers [17]. The available instruments (Table 1) often measure generalized apathy in living with illness or in healthy conditions, and none of them examine the patient’s specific feelings, thoughts, and behaviors in facing chronic illness. Although Geun Seo Jong et al. (2017) used the AES-Self (AES-S)-Self (AES-S) version of Marin’s apathy evaluation scale (AES) to examine the apathy in epileptic patients [12], the AVS does not explicitly examine the apathy related to different aspects of epilepsy. Marin et al. (1991) argue that epilepsy in different groups is under environmental, psychological and social influences that need to be investigated under the same conditions [22]. Because social stigma and chronic nature of epilepsy and its symptoms such as seizures may affect the patient’s feelings, reactions, and behaviour in dealing with the disease and its treatment [33], it is necessary to have a specific instrument assessing apathy in epileptic patients. Collins et al. (2006) emphasized the necessity and appropriateness of using mixed methods to assess existing instruments’ appropriateness or utility [34]. Therefore, this study was conducted to design and psychometrically analyze epilepsy-related apathy scale (E-RAS) in adults with epilepsy.

**Table 1:** Available instruments for assessment of apathy

NO	Authors	Scale Title	Item Number	Domain	Target group	Type
1	Overall and Gorham(1962) [18]	Brief Psychiatric Rating scale	5		Paranoid, Schizophrenic and Depressive	Sub-scale
2	Kay et al.(1989) [19]	Positive and Negative Symptoms scale	8		schizophrenia	Sub-scale
3	Andreasen(1989) [20]	Assessment of Negative Symptoms	8		schizophrenia	Sub-scale
4	Burns etal(1990) [21]	Apathy	5	Single factor	Huntington and Alzheimer disease	full
5	Marin etal(1991) [22]	Apathy Evaluation Scale (AES)	18	3	Well elderly, Left hemisphere stroke, Right hemisphere stroke, Probable Alzheimer’s disease and Major depression	full
6	Cummings et al.(1994) [23]	Neuropsychiatric Inventory	9		dementia patients	Sub-scale
7	Starkstein et al.(1995) [24]	Apathy Scale	14	Single factor	Alzheimer’s disease	full
8	Grace et al.(1999) [25]	Frontal Systems Behaviour scale	27		frontal lobe brain-damaged patients	Sub-scale
9	Strauss and Sperry(2002) [26]	Dementia Apathy Interview and Rating	16		Alzheimer Disease	Sub-scale
10	Norris and Tate(2002) [27]	The Behavioral Assessment of Dysexecutive Syndrome- DEX	20		All groups	Sub-scale
11	Robert et al.(2002) [28]	Apathy Inventory	3	Emotional blunting, Lack of initiative and Lack of interest	Alzheimer’s disease, Parkinson’s disease and mild cognitive impairment (caregiver and patient)	Full
12	Belanger et al.(2002) [29]	Key Behavior Change Inventory	28		Elderly with memory disorder	Sub-scale
13	Sockeel etal(2006) [30]	The Lille apathy rating scale (LARS)	33	reduction in everyday productivity; lack of interest; lack of initiative; extinction of novelty seeking and motivation; blunting of emotional responses; lack of concern; poor social life and extinction of self-awareness	Parkinson’s disease	full
14	Radakovic & Abrahams (2014) [31]	Dimensional Apathy scale (DAS)	24	Executive, Emotional and Behavioral/Cognitive Initiation	neurodegenerative disease and motor dysfunction such as Parkinson’s disease	full
15	Ang etal(2017) [32]	Apathy Motivation Index	18	behavioral, social and emotional	All groups	full

## Methods

### Design and setting

This study of sequential exploratory mixed methods design was conducted in Iran from April 2019 to December 2019.This study is part of a research project entitled “ relationship between disease– related fear with apathy and nutrition status in adults with epilepsy: a multiple-center study(Cod; IR.MUBABOL.HRI.REC.1398.132)”. Creswell and Plano Clark (2011) recommended using sequential mixed methods research design for scale development and exploratory instrument design. Sequential mixed methods research design consists of three phases: a qualitative phase to define the construct of the instrument; an instrument development phase including item generation and revision; and a confirmatory quantitative phase for instrument testing [35]. Hinkin et al. (1995) proposed three phases: item development, scale development, and scale evaluation, to create a rigorous scale [36]. The present study was also conducted in three phases.

### Item generation

The item generation step is also called question development. Two methods, i.e., inductive and deductive, are used to identify appropriate items [36].

### First phase

In this phase, the inductive method was used. The method is also known as grouping or classification from below. In this phase, the items are generated from qualitative data from direct observations and individual interviews or focus groups, including the target-population [37].

### Data collection

The research settings were the Iranian Epilepsy Association and the neurological clinics of the hospitals affiliated to Iran University of Medical Sciences, and the office of neurologists in the cities of north Iran. In this phase, sampling was purposeful. Inclusion criteria included having suffered from epilepsy for at least 1 year, depressive disorder (obtaining a score of 4 or less from the short form of the Beck’s Depression Inventory ([38], treatment with antiepileptic drugs for at least 1 year, age of 74–18 years and lack of substance abuse. Exclusion criteria included unwillingness to continue participation in the study. Participants were people who could provide first-hand information to the researcher (young patients with epilepsy). Sampling continued until data saturation was achieved. Data saturation in qualitative research is obtained when the data are duplicated, and no new code is obtained [39]. Finally, 17 semi-structured face-to-face interviews were performed. Individual interviews lasted 40 min on average. Participants were asked the following questions:

1. Please explain to me the concept of epilepsy-related apathy.

In addition, during the interviews, what are the probing questions such as “what do you mean?”, “If possible, please explain more?” or “How did you feel about that?” were also raised.

At the completion of each interview, participants were asked to state something if they felt it as having not talked of throughout the interview and then the interviewer spoke of the possibility of further interviews. The interviews were conducted in the researcher’s room at the request of the participants.

### Trustworthiness of data

In this study, Guba and Lincoln’s four criteria, i.e., credibility, dependability, confirmability, and transferability, were used to ensure the qualitative phase’s accuracy and precision [40]. To obtain valid data, member checking was used to verify the accuracy of the extracted data and codes. Codes that did not reflect the views of the participants were modified. In order to conduct peer checking, the texts of some of the interviews and extracted codes and categories were reviewed by three faculty members in addition to the authors, with 93–95% agreement among the results. The method proposed by Paulite and Hangler was used to calculate the agreement [41]. In order to investigate the transferability, the findings were shared with some patients with epilepsy who did not participate in the study, and they confirmed the appropriateness of the findings with their experiences. Maximum variation sampling in terms of age, sex, education level, marital status, age at onset of epilepsy, duration of of epilepsy, seizure frequency per month, and duration of administered antiepileptic drugs (AEDs) intake was observed.

### Data analysis

Data analysis was performed using conventional content analysis method based on Graneheim and Lundman method [42]. For this purpose, first, the data were read line by line, and the open codes (which are the words of the participants themselves) were extracted. The obtained codes were compared with previous codes and codes that were conceptually similar were assigned to the same category. Gradually, categories were formed. The categories were also compared and merged with each other as needed, or in some cases, one category was divided into two or more categories, or a code was transferred from one category to another. Eventually, the main subcategories were formed. In this study, MAXQDA/10 software was used to organize and categorize the extracted codes.

### Second phase

In this phase, the deductive method was used. The available literature and scales were reviewed and evaluated. The deductive method is also known as logical partitioning or classification from above [43]. In this phase, the literature review was conducted in Pubmed, Scopus, Web of Science and PsycINFO databases using the keywords fear, apathy, epilepsy-related apathy, adult scale, questionnaire, and epilepsy from 10th April until 1st June 2019. In total, 30 relevant articles were retrieved that had been published from 1962 to 2019. Some of the articles addressed the tools that measured apathy (Table 1), and some were qualitative studies on the concept of apathy and its dimensions.

### Item reduction

#### Third phase

Integration of qualitative and literature reviews: providing the pool of items. First, each interview was coded, and similar codes merged into categories and subcategories. Then all the texts, including the available tools, qualitative studies related to the studied construct were coded separately, and then the same codes were formed the categories and subcategories. The codes and categories extracted from each phase of the study were examined separately. Then all the codes and categories of both phases were put together. Duplicate items were deleted, and similar items merged. Because the codes and subcategories were the basis for building items pool, they were rated more deliberately. They were coded, classified, and labelled over 3 months until the research team reached a consensus. In this part of the study, using the information obtained in the qualitative stage (interviews), a pool of 29 items was formed. An example of the items pool formation process is presented in Table 2. In the deductive stage (literature review), 15 items were obtained and added to the items pool formed in the qualitative stage. The items were re-checked by the research team, the duplicate items were deleted, and similar items were merged. Some items were also modified. The items were edited to be suitable for both low and medium literacy levels. E-RAS was finally included in 31 items for adults with epilepsy.

**Table 2:** An example of the process of determining the spheres designed from participants’ experiences

Item	Category	Participants’ experiences
Criticizing and rejecting me by others reduces my motivation to treat my illness.	Motivation	*I remember going to school. The time I was 15 years old. My friends didn’t let me join their circles. They rejected me. But I hoped I would be cured and my illness would be controlled. I did not lose my spirit. I had motivation for the future of my life.*
I actively follow behaviors related to the dimensions of controlling my illness (such as preventing possible injuries during seizures and adhering to the therapeutic regimen).	Self-Regulatory	*It is important for me to try to follow what my doctor tells me. I don’t ride a bicycle and I care not to get injured during a seizure.*
Cooperation of others, I will explain the conditions/symptoms of my illness to them.	Cognition	*When I meet someone like new colleagues or friends who do not know my condition, I tell them about the disease and its symptoms. I ask them to understand me and help me during the seizure.*
Deprivation of social rights due to my illness has made me angry and frustrated me in continuing my social activities.	Emotional- Effective	*I have long been looking for a job, but as soon as the employer understands that I have epilepsy, he doesn’t give me the job. I wanted to marry my favorite girl, but my illness prevented her family from agreeing. I’m nervous and desperate to do something.*

### Scale evaluation

In examining the validity of a research tool, face validity, content validity, and construct validity need to be evaluated [44].

#### Face validity

The face validity of E-RAS was investigated in two quantitative and qualitative ways:

#### Qualitative face validity

Ten adult patients with epilepsy were asked to comment on each statement’s level of difficulty, appropriateness, and ambiguity. Corrective comments were applied to the instrument. The time required to respond to the tool was also estimated.

#### Quantitative face validity

The E-RAS face validity was quantified using the Item Impact method. For this purpose, for each of the toolbars, a 5-part Likert scale (perfectly important = 5, important = 4, moderately important = 3, slightly important = 2 and not at all important = 1) was considered. Then, using the Item Impact method formula, the quantitative face validity was calculated.

$$ \Big(\mathrm{Importance}\ \left(\%\right)\times \mathrm{Frequency}=\mathrm{Item}\ \mathrm{Impact}\ \mathrm{Score} $$

A score higher than 1.5 was considered acceptable for each item [45].

#### Content validity

Two qualitative and quantitative methods are used to determine the content validity of designed tools [46]. In this study, the content validity of E-RAS was evaluated by two methods: qualitative and quantitative:

#### Qualitative content validity

The qualitative content validity of E-RAS was evaluated by ten experts (5 Nursing PhD holders, two psychologists, two neurologists and three geriatricians). These individuals were asked to comment on the observance of the grammar, the use of appropriate words, the placement of the items in their proper place, and the appropriate scoring of the questionnaire [47]. In this study, Content Validity Ratio (CVR) and Content Validity Index (CVI) were measured to quantify the content validity:

##### CVR

Lawshe’s model (1975) was used to calculate the CVR [48]. In this way, the questionnaire was given to 10 people (the same people who were invited to collaborate to check the validity of quality content), and they were asked to comment on the necessity of tool items using the 3-point Likert scale (Unnecessary = 1, Useful but unnecessary = 2 and Necessary = 3). The CVR was then calculated using the following formula.

$$ \mathbf{CVR}\kern0.5em =\kern0.5em \left(\frac{\mathbf{ne}-\left(\mathbf{N}/\mathbf{2}\right)}{\mathbf{N}\kern0.5em \mathbf{2}}\right)\kern0.5em \mathbf{CVR}\kern0.5em =\kern0.5em \left(\frac{\mathbf{ne}-\left(\mathbf{N}/\mathbf{2}\right)}{\mathbf{N}\kern0.5em \mathbf{2}}\right) $$

The minimum acceptable value for CVR according to the views of 10 experts is 0.62 [49].

##### CVI

To calculate the CVI, the designed tool was given to 10 professionals (the same people who were invited to collaborate to review the CVR tool) to calculate each item based on the Waltz and Basel content index (52) in terms of relevance to a 4-point Likert scale (Irrelevant = 1, Needing major revision = 2, Relevant yet needing revision = 3, and Absolutely relevant = 4). CVI calculation was done by the following formula:

$$ CVI=\frac{ni}{n} $$

where ni represents the number of experts that scored the item as 3 or 4; and n represents the total number of experts panel members.

#### Construct validity

Then the mean CVI was calculated for all tool items; the acceptable value for CVI is equal to and higher than 0.78 [49, 50]. The validity of the structure was assessed using exploratory factor analysis (EFA; *N* = 360) and confirmatory factor analysis (CFA; *N* = 200).

##### EFA

The internal correlation of the instrument should be examined before sampling for construct validity investigation. A pilot study was conducted to calculate it. After confirming the items’ internal correlation, exploratory factor analysis was performed to determine whether the tool is a single scale or consists of several domains. Then, confirmatory factor analysis was performed to confirm the extracted dimensions [51]. Before the EFA, 360 adult patients with epilepsy were enrolled in the study using the Convenience Sampling method. The inclusion and exclusion criteria of the study were the same as those mentioned in the qualitative phase. For this purpose, a cross-sectional study was performed.

To extract latent factors, exploratory factor analysis was performed using the principal axis factoring (PAF), Varimax rotation, and scree diagram. Eigenvalue was used more than once to determine the number of the factors extracted [52]. The Kaiser-Meyer-Olkin (KMO) index was used to assess sampling sufficiency, and the Bartlett’s test of sphericity was used to investigate the appropriateness of the factor analysis model. KMO values between 0.7 and 0.8 are considered acceptable, and values between 0.8 and 0.9 excellent [53].

The presence of one item in the factor was determined based on the following formula, which was obtained approximately 0.3:

$$ \mathrm{CV}=5.152\div \sqrt{\left(n-2\right)} $$

In this formula, CV is the number of factors that can be extracted and n the sample size of the study [54].

##### CFA

The factors extracted using the first- and second-order CFA (maximum likelihood estimation) and the most common goodness-of-fit indices of modeling structural equation were examined. The number of samples in the confirmatory factor analysis was 200 people. Fit indices used in the study include: Chi-square (χ^2^), Chi-square/degree-of-freedom ratio (normalized Chi-square CMIN/DF), Adjusted Goodness-of-Fit Index (AGFI) > 0.8, Parsimonious Comparative Fit Index (PCFI) > 0.50, Comparative Fit Index (CFI) > 0.90, Incremental Fit Index (IFI) > 0.90, Parsimonious Normed Fit Index (PNFI) > 0.50 and Root Mean Square Error of Approximation (RMSEA) < 0.05 good [55]. In the second-order CFA, it was assumed that the extracted latent factors were present in the first-order CFA. Therefore, the second-order CFA, more general concepts at the secondary and higher levels will present. Moreover, the construct validity was investigated through correlations between the construct factors and demographic and clinical variables.

#### Convergent and divergent validity

The convergent and divergent validity of the D-RAS were evaluated based on the Fornell and Larker (1981) approach using Average Variance Extracted (AVE), Maximum Shared Squared Variance (MSV), Average Shared Square Variance (ASV) and Composite Reliability (CR) [56]. Acceptable indicators for convergent validity are AVE > 0.5 and for divergent validity are MSV < AVE and ASV < AVE [57].

### Reliability

In the reliability study, three characteristics internal consistency, stability and error measurement were evaluated:

### Internal consistency

Internal consistency refers to the homogeneity of variables within a tool and, in fact, an estimate of the correlation between the variables that make up the structure or tool [58]. In this study, the coefficients of Cronbach’s alpha coefficient, McDonald’s Omega and Theta (θ) were estimated and values greater than 0.7 were accepted [59]. Then, the CR was calculated using confirmatory factor analysis. By replacing Cronbach’s alpha coefficient in structural equation modelling, we can calculate the construct reliability. The construct reliability can be calculated based on the composite reliability and the Average Variance Extracted (AVE). Construct reliability should fulfil CR > 0.7 and AVE > 0.5 [60].

### Stability

The D-RAS was administered twice with a two-week interval to 50 adults with epilepsy who fulfilled the criteria to enter the study. Pearson correlation coefficient and Intraclass Coefficient Correlation (ICC) correlation coefficient were then calculated. The ICC of 0.8 or higher indicates acceptable stability [61]. During the test-retest, the amount and management of the missing values were taken into account. Another point that was considered was the samples’ stability in the test-retest interval in terms of the characteristic in question. In case of any mental, psychological or severe stress, the sample was excluded from the second phase.

### Standard error of measurement (SEM)

SEM is one of the indices of measurement accuracy and test reliability. Due to the error in repeating each measurement, there is always some difference [62]. In the present study, the standard error measurement ($\mathrm{SEM}\kern0.5em =\kern0.5em \mathrm{SD}\kern0.5em \times \kern0.5em \sqrt{1- ICC}\kern0.5em \times \kern0.5em \sqrt{1- ICC}$) and the minimally detectable change ($\mathrm{MDC}=\mathrm{SEM}\times Z\kern0.5em score\kern0.5em \times \kern0.5em \sqrt{2}\times Z\kern0.5em score\kern0.5em \times \kern0.5em \sqrt{2}$) and the minimally important change (**MIC** ***=*** **0.5** ***× SD of∆score × SD of∆score***) were calculated.

### Ceiling effect and floor effect

This effect occurs when more than 15% of the respondents score the highest or lowest attainable score [51].

### Scale scoring

In this instrument, the Likert scale was used for responding to the items. In the final version of the questionnaire, standardization 100 method was used to score and compare the scores of different subscales of the questionnaire. The following linear transformation formula was used to convert the scores of the subscales and the whole questionnaire to a score of 0 to 100 [63].

$$ \mathrm{transformed}\kern0.5em \mathrm{score}=\frac{actual\kern0.5em raw\kern0.5em score- lowest\kern0.5em possible\kern0.5em raw\kern0.5em score}{possible\kern0.5em raw\kern0.5em score\kern0.5em range}\times 100 $$

#### The normal distribution of data, outliers and missing data

In order to determine whether data distribution is normal or not, skewness and kurtosis indices should be calculated. The assumption of normality was investigated on the basis of skewness at ±3 and kurtosis at ±7 [64]. Mardia coefficient (8>) was used to check the normality of multivariate normality [65]. To investigate the lack of multivariate outlier data, the d-squared Mahalanobis index (above 20) was examined (*P* < 0.001) [66]. The percentage of missing data was evaluated using Multiple Imputation and then replaced by the average respondent response [57].

### Data analysis

For EFA, SPSS 24 software was used, and for CFA, AMOS24 software, and for other calculations, EXCEL 2016 software was used. JASP software was also used in this study to calculate the Omega McDonald’s coefficient. Depending on the type of variable, Pearson’s correlation coefficient or point-biserial and polyserial correlation coefficients were used to investigate the correlation between factors and demographic and clinical variables.

## Results

The demographic characteristics of the study participants are presented in Table 3.

**Table 3:** Socio-demographic and clinical profiles of the participants (***N*** = 560)

Variable	N	%
**Gender**		
**Female**	306	54.6
**Male**	254	45.4
**Education level**		
Illiterate	57	10.2
Under high school diploma	265	47.3
High school diploma	187	33.4
Academic education	51	9.1
**Marital status**		
Single	324	57.9
Married	204	36.4
Widow/widower	32	5.7
**Occupation**		
Civil servant	37	6.6
Laborer	83	14.8
Retired	13	2.3
Jobless	246	43.9
Self-employed	60	10.7
Housewife	121	21.6
**Income level**		
Sufficient	98	17.5
Approximately sufficient	214	38.2
Lower than sufficient	248	44.3
**Insurance**		
Yes	460	82.1
No	100	17.9
**Intensity of physical activity**		
Low	164	29.3
Moderate	309	55.2
Intense	87	15.5
**BMI**		
< 19	24	4.3
19–21	68	12.1
22–23	203	36.3
> 23	265	47.3
**Age: mean (SD), Years**	38.83 (11.78)	
**Age at onset of epilepsy: mean (SD), years**	7.97 (7.58)	
**Seizure frequency: mean (SD), month**	2.53 (2.55)	
**Duration of epilepsy: mean (SD), years**	30.70 (11.49)	
**Duration of administered antiepileptic drugs (AEDs) intake: mean (SD), years**	27.46 (11.45)	

**Fig. 1:**
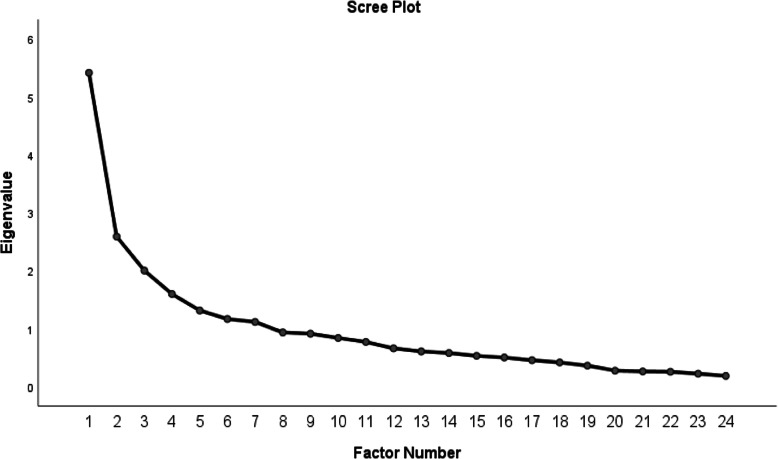
Scree plot for the exploratory factor analysis of the E-RAS

In the qualitative section of the face validity investigation, the item I disagree with most of the suggestions of my treatment team was deleted. In the quantitative section of the face validity investigation, two items: Looking at my job or education is important to me to the end and I have the initiative were deleted due to score of less than 1.5. Therefore, in this section, the 31-item scale was reduced to a 28-item scale. In the qualitative study of content validity, two items were modified, and all the proposed changes of experts were made to the appearance of items. The quantitative study of content validity was performed by CVR and CVI, with one item “I’m concerned about my illness” was deleted due to a CVR of less than 0.62 and one item “I don’t care about communicating with the treatment team” due to a CVI of less than 0. 78. Finally, a 26-item scale remained to be investigated for its construct validity. The results also showed that KMO obtained 0.728 and Bartlett’s test obtained 3154,373 (*P* < 0.001). The scree diagram (Fig. 1) shows that four factors were extracted from the exploratory factor analysis of the E-RAS construct. These four latent factors accounted for 3.632, 3.162, 2.866, and 1.944 of the Eigenvalue, respectively, collectively explaining 48,351% of the total variance in the E-RAS construct. The results of the exploratory factor analysis showed that the two items Although I’m sick, I’m neither happy nor sad (I’m something in the middle) and Worrying about my illness has overshadowed my emotional reactions were deleted because of having a factor load of less than 0.4 (Table 4).

**Table 4:** Exploratory factor analysis of the E-RAS (*N* = 360)

Factor Name	Items	Factor loading	^**a**^h2	Eigenvalues	% of variance
**Motivation**	2. Despite having epilepsy, it’s easy for me to pursue my interests/aspirations.	0.648	0.530	3.632	15.134
	I’m interested in engaging in NGOs of epileptic patients.	0.609	0.424		
	21. To start any new therapeutic method or recommendation, I need a force to motivate me.	0.424	0.391		
	17. Someone needs to listen to me every day about what I can do to manage my illness.	0.594	0.475		
	9-I need energy to follow up my illness	0.553	0.609		
	3. Criticizing and rejecting me by others reduces my motivation to treat my illness.	0.572	0.355		
	26. I’m not interested in participating in self-care programs.	0.592	0.384		
**Self-Regulatory**	I assess how to do health-promoting behaviors (such as exercising, resting adequately, eating healthy foods, avoiding alcohol, smoking and drugs, and avoiding stress).	0.662	0.547	3.162	13.174
	10. I actively follow behaviors related to the dimensions of controlling my illness (such as preventing possible injuries during seizures and adhering to the therapeutic regimen).	0.618	0.574		
	5. I believe I can actively participate in decisions related to disease management.	0.563	0.407		
**Cognition**	11. I understand the importance of self-care.	0.595	0.638	2.866	11.940
	13. I know that I have to follow my treatment protocol for the rest of my life.	0.794	0.640		
	14. I understand the symptoms and consequences of my illness (such as seizures, occupational, educational, and family problems, and cognitive problems such as time, place, and person, and memory problems).	0.785	0.650		
	16. I know I need to follow up my treatment on time and not delay it.	0.484	0.536		
	15. To justify and attract the cooperation of others, I will explain the conditions/symptoms of my illness to them.	0.488	0.373		
**Emotional- Effective**	25. I don’t care how others communicate with me.	0.630	0.475	1.944	8.102
	6. The new goals and plans I have for the future of my life; are not overshadowed by my illness.	0.791	0.634		
	12. In controlling my illness, I accept the new methods offered by the treatment team (such as brain surgery and traditional medicine).	0.568	0.376		
	18. Deprivation of social rights due to my illness has made me angry and frustrated me in continuing my social activities.	0.515	0.376		
	19. The uncertainty about the future of my illness has made precautions related to treatment unimportant to me.	0.657	0.501		
	23. I don’t get excited when I have positive treatment results.	0.645	0.437		
	4. Although I suffer from distress, I am interested in expressing my feelings about my illness.	0.514	0.354		
	20. My fear of the symptoms of the disease has led me toward feeling a kind of alienation.	0.653	0.585		
	7. I don’t care how others react to my symptoms.	0.594	0.534		

^a^h^2^: Communalities

**Fig. 2:**
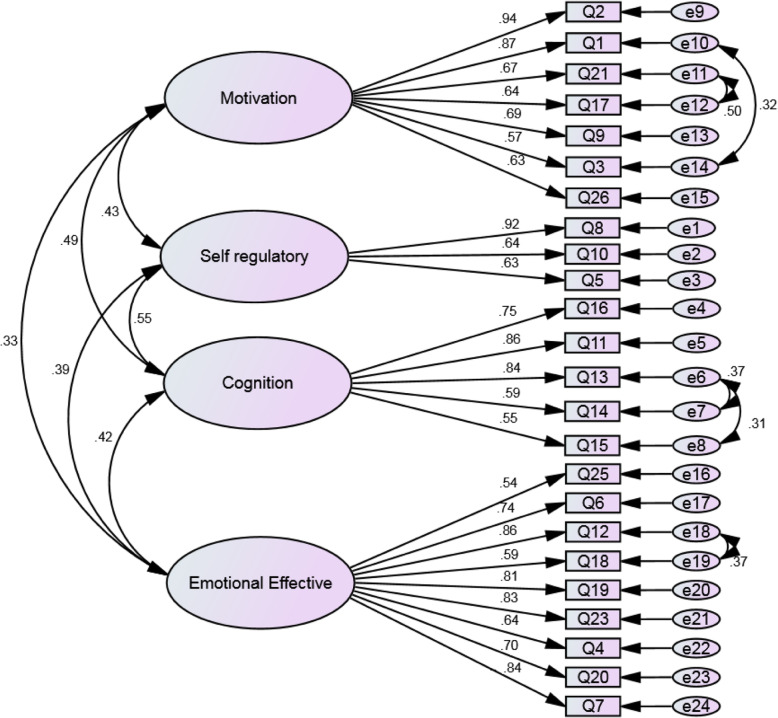
Structure of E-RAS: modified model of first-order confirmation factor analysis

**Fig. 3:**
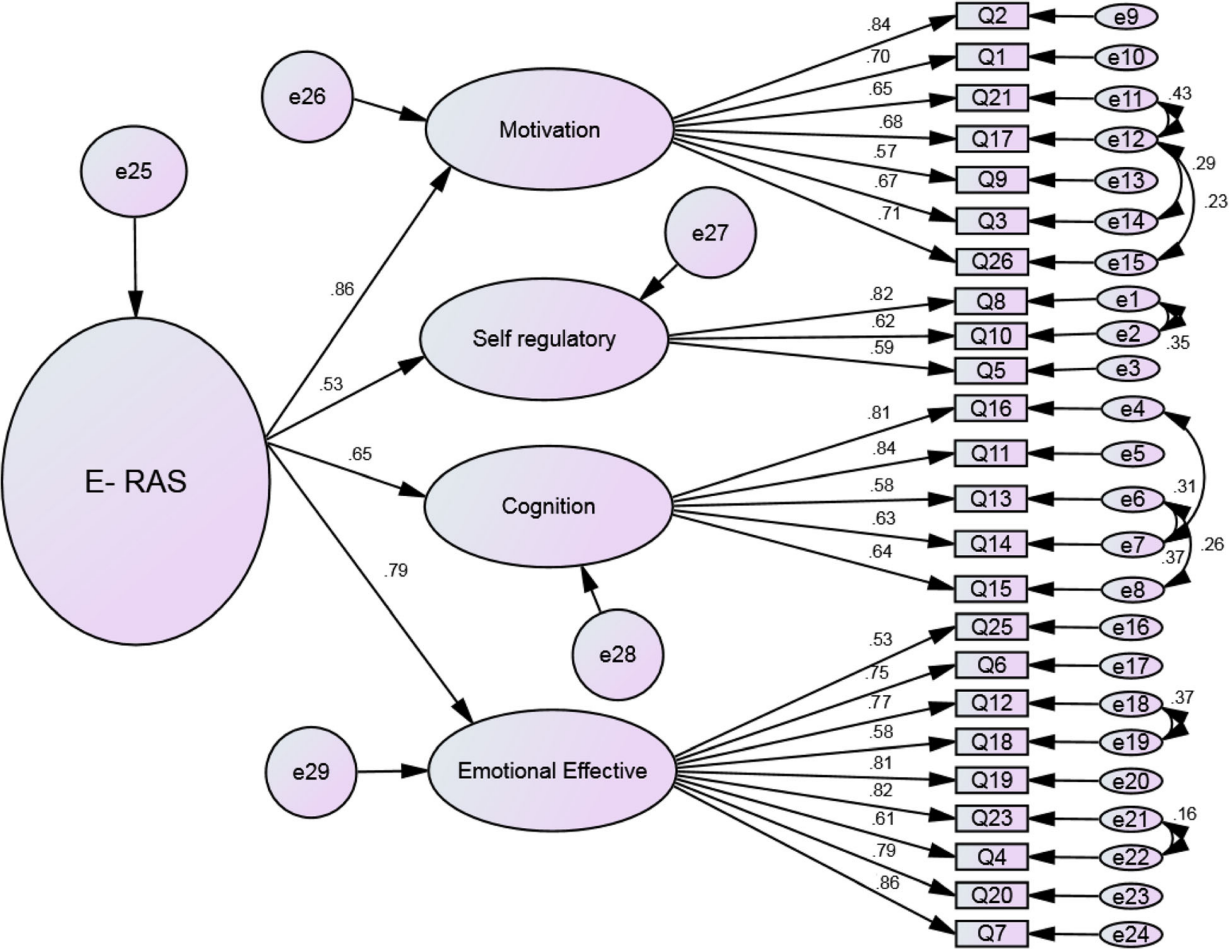
Structure of E-RAS: modified model of second-order confirmatory factor analysis

In the first-order factor analysis, the goodness of fit index (chi-square) was obtained 577.195 (*P* < 0.001) (241) χ2. Then, to evaluate the fit of the model, other indicators were examined that all indicators RMSEA = 0.075, PCFI = 0.64, PNFI = 0.68, AGFI = 0.70, IFI = 0.92 and CFI = 0.91 confirmed the appropriate fit of the final model (Table 5 and Fig. 2). After study of the first-order confirmatory factor analysis, the E-RAS structural components were separately investigated for correlation between the structures and the subscales were identified using the structural equation model to measure whether the number of components is incorporated into the general E-RAS concept, The second-order factor analysis was also performed. The fit indices of this confirmatory factor analysis are shown in Table 5 in comparison with those of the first-order confirmatory factor analysis. Figure 3 illustrates the structural model and confirmatory factor analysis of the E-RAS construct with factor loads with standardized coefficients. The values of the factor load obtained for all E-RAS items were higher than 0.50 (*P* < 0.001).

**Table 5:** Fit indices of the first- and second-order confirmatory factor analysis of the E-RAS

CFA	χ^**2**^	df	***P***-value	CMIN/df	RMSEA	PCFI	PNFI	AGFI	IFI	CFI
**First-order after structure modification**	577.195	241	< 0.001	2.39	0.075	0.64	0.68	0.70	0.92	0.91
**Second-order after structure modification**	480.868	239	< 0.001	2.01	0.062	0.72	0.83	0.88	0.95	0.96

*Abbreviations*: *E-RAS* epilepsy– related apathy scale, *CFA* confirmatory factor analysis, *CMIN/DF* Chi-square/degree-of-freedom ratio, *RMSEA* Root Mean Square Error of Approximation, *PCFI* Parsimonious Comparative Fit Index, *PNFI* Parsimonious Normed Fit Index, *AGFI* Adjusted Goodness-of-Fit Index, *IFI* Incremental Fit Index, *CFI* Comparative Fit Index

Fit indices: PNFI, PCFI, AGFI (> 0.5), CFI, IFI (> 0.9), RMSEA (> 0.08), CMIN/DF (> 3 good, > 5 acceptable)

The results show that epilepsy-related indifference in all four factors had a significant relationship with the demographic and clinical variables of adults with epilepsy (Table 6).

**Table 6:** Correlations of the E-RAS subscale with demographic/disease-related variables

Demographic/disease Variables	E-RAS subscales			
	Motivation	Self-regulatory	Cognition	Emotional-Effective
**Age**	.252**	.206**	.145**	.153**
**Age at onset of epilepsy**	.160**	−.132*	−.108*	.122*
**Seizure frequency**	.173**	−.129*	−.147*	−.161**
**Level of education**	.154*	.219**	.118**	.134**
**Duration of AEDs intake**	.187**	.277**	.146**	.129*
**Gender**^**b**^	−.169**	−.225**	−.191**	−.261**
**Mariage**^**a**^	−.199**	−.114*	−.104*	−.168**

^a^Polyserial correlations

^b^point-biserial correlation

*p < .05

**p < .01

The results also showed that, in the first-order confirmatory factor analysis, the AVE of all factors was greater than 0.5 and the AVE of each factor was greater than that of ASV and MSV. The results showed that the E-RAS construct had convergent and divergent validity. In the second factor analysis, AVE was obtained > 0.5, which confirms the convergent validity (Table 7).

**Table 7:** Convergent and divergent validity, internal consistency, and constructs reliability of E-RAS

Factor	***α***	θ	Ω	CR	First-order			Second-order
					AVE	MSV	ASV	AVE
**Motivation**	0.770	0.726	0.728	0.883	0.528	0.240	0.177	0.516
**Self-Regulatory**	0.763	0.759	0.742	0.780	0.550	0.302	0.213	
**Cognition**	0.792	0.803	0.815	0.846	0.531	0.302	0.239	
**Emotional-Effective**	0.889	0.846	0.840	0.912	0.541	0.176	0.145	

*Abbreviations*: *E-RAS* epilepsy– related apathy scale, *a* Cronbach’s alpha coefficients, *θ* theta coefficient, *Ω* McDonald omega coefficient, *CR* construct reliability, *AVE* average variance extracted, *MSV* maximum shared squared variance, *ASV* average shared squared variance

The results also showed that the internal stability and CR (> 0.7) of the four extracted factors from the E-RAS construct were confirmed (Table 7). The stability (test-retest) of the scale was investigated using ICC. The mean pre- and post-test scores were 64.46 ± 10.96 and 62.25 ± 6.48, respectively. The ICC was equal to 0.843 (*P* < 0.001, 95% CI: 0.773–0.900) (Table 8). The results showed that the SEM, MDC and MIC of E-RAS were 4567, 12,660 and 5321, respectively (Table 8).

**Table 8:** ICC, SEM, MDC and MIC of the E-RAS in adult with epilepsy

Factor	Range of score	ICC(95% CI)	***P***-value	SEM	MDC	MIC	Agreement
**Motivation**	12–28	0.707(0.614–0.853)	< 0.001	2.138	5.926	1.472	positive
**Self-Regulatory**	6–12	0.798(0.696–0.898)	< 0.001	.822	2.279	0.287	positive
**Cognition**	5–20	0.843(0.762–0.903)	< 0.001	1.434	3.975	1.813	positive
**Emotional-Effective**	17–34	0.750(0.684–0.879)	< 0.001	2.305	6.389	2.952	positive
**Total**	45–91	0.843(0.773–0.900)	< 0.001	4.567	12.660	5.321	positive

*Abbreviations*: *ICC* intra-class correlation, *SEM* standard error of measurement, *MDC* Minimal Detectable Change, *MIC* minimal important change

The results showed that more than 15% of the respondents obtained the highest or lowest possible score on E-RAS (Table 9).

**Table 9:** Percentage of people who scored the minimum and maximum scores on each subscale and the entire E-RAS scale

Factor	Range of variations	Minimum score (%)	Maximum score (%)
**Motivation**	26–10	22.25	57.2
**Self-Regulatory**	12–3	21.91	54.9
**Cognition**	20–8	28.16	52.13
**Emotional-Effective**	30–13	26.35	48
**Total**	76–48	23.41	53.03

### Scale scoring

The final version of E-RAS consists of 24 items. Scale includes 4 dimensions including motivation (7 items); self-regulatory (3 items); cognition (5 items) and emotional-effective (9 items). The items are rated on a 4-part Likert scale (Almost always = 4, Often = 3, Occasionally = 2, Hardly Ever = 1). The items 17,18,19,20, 21, 23, 25 and 26) are scored inversely. The minimum and maximum attainable scores on the scale are 45 and 91, respectively. The responses to each subscale’s items are summed up, and then calculated and expressed as percentage for each subscale and the entire scale using the linear transformation formula. Eventually a score of 0 to 100 is obtained, with a lower score indicating less epilepsy related apathy in an adult patient with epilepsy.The results showed that the value of Mardia coefficient is 8.54 and its critical ratio is 2.46. Therefore, it can be concluded that the hypothesis of the multivariate normality with proper approximation was fulfilled.

## Discussion

This study investigated the reliability and validity of the E-RAS, a new instrument for the assessment of apathy in adults with epilepsy. Available tools such as dimensional apathy scale measure apathy in healthy and normal samples [67] and other tools (Table 1) measure general apathy in different target groups. Therefore, the researchers preferred not to compare the psychometric properties of E-RAS with other tools that measure apathy. The present study was conducted with the aim of designing and evaluating E-RAS by mixed method. Recently, researchers have presented mixed methods as the most appropriate method for validation. The use of quantitative and qualitative methods for the generation of items increases the validity of the content [68]. Onwuegbuzie et al. (2010) also believe that mixed methods can be used to provide content-related evidence for face validity, item validity and sampling validity, and construct-related evidence for substantive validity, outcome validity, and generalizability [69]. In this study, inductive and deductive methods were used to prepare items pool. When the purpose is to design a new tool or develop a scale, the validity and reliability of the item generation phase can be increased by using qualitative and quantitative methods [35, 70]. In the present study, after preparing the primary instrument, E-RAS psychometric indicators were examined. Designing or selecting research instruments requires special attention to psychometric criteria [71]. In this study, in order to investigate the face validity of E-RAS, the opinions of a number of patients with epilepsy were elicited. Target population’s judgements are extremely important in assessing face validity and can make the tool applicable to the target group [72]. In this study, two qualitative and quantitative methods were used to investigate the content validity. The use of ideas of experts with knowledge and experience in the subject matter can significantly help to increase the content validity of new tools [73]. Construct validity can be provided by factor analysis, testing hypothesis, and convergent and divergent validity [62], all of which were investigated in the present study. In the present study, the Meyer-Kaiser-Olkin (KMO) index was examined before performing the exploratory factor analysis to examine sampling adequacy. The amount of KMO = 0.8 indicates that the number of samples is sufficient [74]. Bartlett’s test of sphericity was also run to investigate the appropriateness of factor analysis. The significance of this test means that the correlation matrix between the items is confirmed and the factor analysis model is appropriate [75]. In the present study, 360 samples were used in the investigation of exploratory factor analysis and 200 samples in study of confirmatory factor analysis. Determining the number of samples is essential for factor analysis. Costello and Osborne (2005) consider the best way to determine sample size to be the ratio of sample to item. They believe that it is better to take 10 or 20 samples for each item [76]. According to the obtained results, one of the best fit indexes of the equations models is the root mean error of approximation (RMSEA). For models with a good fit, this value should be less than 0.09 [77]. Given the value of the RMSEA in the present study, the results indicated that the model was appropriate. The results of exploratory and confirmatory factor analyses showed that E-RAS has four dimensions, namely, motivation, self-regulatory, cognition, and emotional-effective. Sockeel et al. (2006) stated that apathy has four dimensions including intellectual curiosity, self-awareness, emotion and action initiation [30]. The first dimension is motivation, which explains 15,134% of the total variance. This dimension addresses issues such as the patient’s motivation and interests in disease management. Ang et al. (2017) labelled this dimension as social motivation and argued that this subscale includes items that examine a person’s motivation to participate in social interactions [32]. The second dimension is Self-Regulatory, which explained 13.174% of the variance. The items of this dimension address the patients’ value-based behaviours and efforts aimed to control the situation. This dimension was labelled in the study of Levy and Dubois (2006), quoted by Habib (2004), as auto-activation and defined as a low desire toward thoughts and related behaviours such as lack of motor responsiveness (akinesia) and lack of discourse [78]. Ang et al. assigned the behavioural activation label to this subscale. Ang argues that this dimension examines things like self-initiate goal-directed behaviour (for example, what a person should do without the need to others’ reminding). However, in the present study, such variables were assigned to the motivation dimension. The third dimension is cognition, that explained 11.940% of the variance in the epilepsy-related apathy variable in our study. In fact, this dimension includes the items that address the patient’s inability to understand and recognize the disease, its consequences, and treatment protocol. In their study, Levy and Dubois (2006) also considered cognition as one of the dimensions of apathy. According to Levy, this dimension addresses an individual’s inability to manage cognitive goals and strategies with a negative impact on cognitive and action planning [78]. The fourth dimension is emotional-effective. This dimension was found to explain 8.102% variance in the epilepsy-related apathy variable. This dimension measures the patient’s emotional and behavioural reactions in facing others’ reactions to the disease and its symptoms, as well as the patient’s reaction to the disease and its complications. This dimension was also found as one of the dimensions of apathy in the study of Levy and Dubois (2006) [78]. Ang et al. (2017) later labelled this dimension as emotional sensitivity and argued that this subscale included items that express a person’s positive and negative emotions, which seem to be similar to emotional blunting. The results show that epilepsy-related apathy in all four factors had a significant relationship with the demographic and clinical variables of adult epileptic patients [32].

The results also showed that there was a positive correlation between the four E-RAS subscales and older age. Reasonable assumption is that aging can be a factor for reduced motivation and hope in patients with epilepsy to adopt a new plan for the future, and also decreased motivation to adhere to treatment. The results also showed a positive correlation between E-RAS subscales and long-term administration of antiepileptic drugs (AEDs). Prolonged use of AEDs appears to cause fatigue and frustration with the treatment protocol. Therefore, the patient’s motivation to pursue the goals of the treatment protocol is reduced. The study of Seo et al. (2017) also showed that there was a statistically significant relationship between the duration of AEDs consumption and apathy [12]. The results of the present study showed that the rate of epilepsy-related apathy reduced with increasing number of seizures in most subscales. Besides that, apathy in females and married people is less than other groups. It can be argued that increasing the number of seizures increases the perception of the threat and increases the patient’s motivation to adhere to a treatment protocol and increases setting goals to achieve recovery and to reduce the effects of the disease. Married people seem to be more motivated to manage themselves because of their social support. The results also showed that women had less apathy than men. This may be due to the characteristics of women and their sensitivity and paying attention to various aspects of health, illness and treatment. In examining the reliability of an instrument, three characteristics, internal consistency, stability, and error measurement are mainly evaluated [44], all three of which were measured in the present study. The alpha coefficient for the whole E-RAS was 0.815. An alpha coefficient of 0.7 is often considered as an acceptable threshold for reliability. However, 0.8 and 0.95 are preferred for the psychometric quality of scales [79]. To test the stability in the present study, the test-retest method was implemented. Stability of an instrument refers to the repeatability of its administration or its reliability. In the test-retest, which is the most common method to test stability, the test is administered twice to one group with a given time interval. To this end, after a period of time (usually 2 weeks), the same instrument is administered again to the same respondents, and then the correlation between the test and retest scores is calculated [62]. Efforts were made to make E-RAS items unambiguous and straightforward so that low-literacy participants could fill out the instrument. Schinka et al. (2013) argue that instrument items should be unambiguous and straightforward, and should not contradict religious beliefs, ethnicity, race, economic status, or gender [80]. Some E-RAS items are scored inversely. Inversely scored items have been proposed as a strategy to prevent response bias in using self-report instruments. Response bias refers to a pattern of response that does not reflect the actual opinions or conditions of the respondents [81]. Missing values and their management were important throughout the factor analysis and should be reported [75]. In this study, the missing values were reported.

## Conclusion

The results of the present study showed that E-RAS was a multidimensional instrument and had acceptable validity and reliability for the study of epilepsy-related apathy symptoms in adult epileptic patients. E-RAS is in fact a measure of motivation and can serve as a valid predictor of epilepsy recovery. E-RAS may be useful in diagnosing the patient’s problems in managing the disease, or it may be used as a guide for families dealing with functional problems of epileptic patients.

## Implications and limitations

- In this study, efforts were made to investigate the validity of a research instrument through a psychometric process and by reporting the relevant details in order to provide appropriate evidence to ensure its validity.
- In designing E-RAS, it was attempted to reduce the number of items so that it would not be boring for patients with epilepsy.
- E-RAS items were modified by experts and patients with epilepsy throughout various phases to ultimately achieve an instrument that can be understood and accepted by samples with different levels of literacy and sociocultural status.
- Sampling in the qualitative and quantitative phases were done in different regions of Iran, enabling us to reduce the effect of the culture variable on samples’ responses.
- In this study, an adequate number of samples were included so that the results could represent epilepsy-related apathy in adults.
- Psychological and environmental variables may be the primary cause of epilepsy. Therefore, the results of the present study may be influenced by variables that have not been taken into account in the current study.
- E-RAS is a self-report instrument and therefore can lead to report errors.

## Supplementary Information

**Additional file 1.** E-RAS (English language version).

## Data Availability

The excel file can be provided on demand. F Gh (corresponding author) should be contacted by anyone requesting the data.

## Abbreviations

E-RAS: Epilepsy– related apathy scale
CVR: Content validity ratio
CVI: Content validity index
KMO: Kaiser-Meyer-Olkin
EFA: Exploratory factor analysis
ICC: Intraclass correlation coefficient
PAF: Principal axis factoring
CMIN/DF: Chi-square degree-of-freedom ratio
χ^2^: Chi-square
AGFI: Adjusted Goodness-of-Fit Index
PCFI: Parsimonious Comparative Fit Index
CFI: Comparative Fit Index
IFI: Incremental Fit Index
PNFI: Parsimonious Normed Fit Index
RMSEA: Root Mean Square Error of Approximation
AVE: Average Variance Extracted
MSV: Maximum Shared Squared Variance
ASV: Average Shared Squared Variance
