# Supplementary material for: Design and psychometric evaluation of epilepsy-related apathy scale (E-RAS) in adults with epilepsy: a sequential exploratory mixed methods design
Source: BMC Neurol. 2021 Mar 17;21:121. doi: 10.1186/s12883-021-02139-2 (PMC7967960; doi:10.1186/s12883-021-02139-2)
Supplement: Supplementary file 1 — Additional file 1. E-RAS (English language version). [file 12883_2021_2139_MOESM1_ESM.docx]

| **NO** | **Items** | Hardly Ever | Occasionally | Often | Almost Always |
| --- | --- | --- | --- | --- | --- |
| 2 | Despite having epilepsy, it's easy for me to pursue my interests/aspirations. |  |  |  |  |
|  | I'm interested in engaging in NGOs of epileptic patients. |  |  |  |  |
| 21 | To start any new therapeutic method or recommendation, I need a force to motivate me. |  |  |  |  |
| 17 | Someone needs to listen to me every day about what I can do to manage my illness. |  |  |  |  |
| 9 | I need energy to follow up my illness |  |  |  |  |
| 3 | Criticizing and rejecting me by others reduces my motivation to treat my illness. |  |  |  |  |
| 26 | I'm not interested in participating in self-care programs. |  |  |  |  |
|  | I assess how to do health-promoting behaviors (such as exercising, resting adequately, eating healthy foods, avoiding alcohol, smoking and drugs, and avoiding stress). |  |  |  |  |
| 10 | I actively follow behaviors related to the dimensions of controlling my illness (such as preventing possible injuries during seizures and adhering to the therapeutic regimen). |  |  |  |  |
| 5 | I believe I can actively participate in decisions related to disease management. |  |  |  |  |
| 11 | I understand the importance of self-care. |  |  |  |  |
| 13 | I know that I have to follow my treatment protocol for the rest of my life. |  |  |  |  |
| 14 | I understand the symptoms and consequences of my illness (such as seizures, occupational, educational, and family problems, and cognitive problems such as time, place, and person, and memory problems). |  |  |  |  |
| 16 | I know I need to follow up my treatment on time and not delay it. |  |  |  |  |
| 15 | To justify and attract the cooperation of others, I will explain the conditions/symptoms of my illness to them. |  |  |  |  |
| 25 | I don't care how others communicate with me. |  |  |  |  |
| 6 | The new goals and plans I have for the future of my life; are not overshadowed by my illness. |  |  |  |  |
| 12 | In controlling my illness, I accept the new methods offered by the treatment team (such as brain surgery and traditional medicine). |  |  |  |  |
| 18 | Deprivation of social rights due to my illness has made me angry and frustrated me in continuing my social activities. |  |  |  |  |
| 19 | The uncertainty about the future of my illness has made precautions related to treatment unimportant to me. |  |  |  |  |
| 23 | I don't get excited when I have positive treatment results. |  |  |  |  |
| 4 | Although I suffer from distress, I am interested in expressing my feelings about my illness. |  |  |  |  |
| 20 | My fear of the symptoms of the disease has led me toward feeling a kind of alienation. |  |  |  |  |
| 7 | I don't care how others react to my symptoms. |  |  |  |  |
